# Stimulated photosynthesis of regrowth after fire in coastal scrub vegetation: increased water or nutrient availability?

**DOI:** 10.1093/treephys/tpae079

**Published:** 2024-07-03

**Authors:** Erin I E Rogers, Kazi R Mehnaz, David S Ellsworth

**Affiliations:** Hawkesbury Institute for the Environment, Western Sydney University, Penrith, NSW, Australia; Hawkesbury Institute for the Environment, Western Sydney University, Penrith, NSW, Australia; Hawkesbury Institute for the Environment, Western Sydney University, Penrith, NSW, Australia

**Keywords:** Banksia, epicormic resprouting, leaf N concentration, leaf P concentration, post-fire recovery, stomatal conductance

## Abstract

Fire-prone landscapes experience frequent fires, disrupting above-ground biomass and altering below-ground soil nutrient availability. Augmentation of leaf nutrients or leaf water balance can both reduce limitations to photosynthesis and facilitate post-fire recovery in plants. These modes of fire responses are often studied separately and hence are rarely compared. We hypothesized that under severe burning, woody plants of a coastal scrub ecosystem would have higher rates of photosynthesis (*A*_net_) than in unburned areas due to a transient release from leaf nutrient and water limitations, facilitating biomass recovery post-burn. To compare these fire recovery mechanisms in regrowing plants, we measured leaf gas exchange, leaf and soil N and P concentrations, and plant stomatal limitations in Australian native coastal scrub species across a burn sequence of sites at 1 year after severe fire, 7 years following a light controlled fire, and decades after any fire at North Head, Sydney, Australia. Recent burning stimulated increases in *A*_net_ by 20% over unburned trees and across three tree species. These species showed increases in total leaf N and P as a result of burning of 28% and 50% for these macronutrients, respectively, across the three species. The boost in leaf nutrients and stimulated leaf biochemical capacity for photosynthesis, alongside species-specific stomatal conductance (*g*_s_) increases, together contributed to increased photosynthetic rates after burning compared with the long-unburned area. Photosynthetic stimulation after burning occurred due to increases in nutrient concentrations in leaves, particularly N, as well as stomatal opening for some species. The findings suggest that changes in species photosynthesis and growth with increased future fire intensity or frequency may be facilitated by changes in leaf physiology after burning. On this basis, species dominance during regrowth depends on nutrient and water availability during post-fire recovery.

## Introduction

Wildfires currently affect up to 500 million hectares of the Earth’s terrestrial surface each year (Shi and Touge 2022), releasing carbon (C) into the atmosphere. The extent, severity, and frequency of wildfires are increasing in many ecosystems (Boer et al. 2020; Canadell et al. 2021; Higuera and Abatzoglou 2021), leaving areas open for degradation, soil erosion and invasive species if rapid plant recovery of extant species does not occur (Lambers et al. 2022). Woody scrub vegetation found along the east coast of Australia is particularly fire-prone (Canadell et al. 2021), with high fire-return frequencies selecting vegetation composition towards a high proportion of resprouting species (Clarke and Knox 2002). Resprouting is efficient at regaining biomass post-fire, so understanding the mechanisms that drive the photosynthetic productivity of the regrowth in resprouting vegetation is especially pertinent. This regrowth has key repercussions for future carbon gain in burned ecosystems and for standing stocks of carbon and nutrients on sites after repeated burns (Bennett et al. 2014; Wright et al. 2021).

By removing aboveground biomass, wildfires affect above-ground carbon stocks but can also affect below-ground resources via fire-induced alterations to soil properties through organic matter removal and ash deposition (Escudey et al. 2010; Schaller et al. 2015; Butler et al. 2018). Depending on the intensity of the fire and soil temperatures reached during burning, fire-affected soils show increases in pH (Certini 2005), reduced soil porosity (Everett et al. 1995; Robichaud 2000; Johansen et al. 2001), increased exchangeable cation concentrations (Khanna et al. 1994; Escudey et al. 2010), and altered nutrient content and availability (Butler et al. 2018; Butler et al. 2019), especially for nitrogen (N) and phosphorus (P). Fire-prone ecosystems on low P soils are particularly susceptible to changes in nutrients, especially P (Dijkstra and Adams 2015), and will experience significant perturbations to nutrient stoichiometry following fire (Butler et al. 2019). Ash deposition from combusted biomass burning following fire adds P in the bioavailable form of orthophosphate to the soil (Cade-Menun et al. 2000; Wang et al. 2015). The process of N and P liberation from biomass and redistribution to the soil as ash is crucial in the post-fire landscape, especially where P is in scarce supply such as for coastal scrub vegetation (Wright et al. 2001; Santin et al. 2018). Vegetation that was previously P-limited can experience a transient increase in P after burning, leading to enhanced plant productivity during recovery (Dijkstra and Adams 2015) and accelerated regrowth.

In addition to soil nutrient and biogeochemical alterations driven by fire, another effect of aboveground biomass burning is the change in plant root:shoot and root:leaf area ratios. Changes to these ratios can elicit strong effects on plant hydraulic function (Ma et al. 2010) and increases in plant water use due to this imbalance. For instance, leaf area is reduced following fire resulting in an increased root:leaf area ratio, which supports higher rates of transpiration in the remaining and resprouting leaves compared with non-fire affected leaves (Nolan et al. 2014). This can translate to higher transpiration rates per unit leaf area and reduced stomatal limitations to photosynthesis resulting in greater rates of photosynthesis in these resprouted leaves (Goorman et al. 2011). The opposite, an observed reduced transpiration after root pruning (Ma et al. 2010), was a response to decreased root:shoot ratio, demonstrating that this effect is opportunistic in plants. These studies highlight the strength of the root:shoot and root:leaf area balance and how alterations to that balance affect transpiration and thus photosynthesis by releasing stomatal limitations. Following fire, resprouters experience an advantage compared with non-sprouters, since root:leaf area imbalances encourage the rapid photosynthetic recovery of burnt species due to changes to plant hydraulic function (Nolan et al. 2014). Thus, despite the importance of nutrient release and lack of water limitations after fire, these processes have not been compared with respect to their impacts on plant regrowth after burning (Clemente et al. 2005; Cernusak et al. 2006; Jorge et al. 2010; Renninger et al. 2013; Nolan et al. 2014), limiting our understanding of ecosystem recovery after fire.

Resprouting is a key functional trait for survival in fire-prone landscapes where persistent disturbance has inflicted a strong selective pressure on vegetation (Pausas and Keeley 2014). In Australia, most post-fire recovery is due to resprouting from epicormic buds (Pate et al. 1990; Clarke et al. 2013; Clarke et al. 2015). The ability of resprouters to recover quickly within a post-fire environment is strongly affected by both nutrient and water availability within the soil (Clarke et al. 2013). As few studies have quantified the strength of increased plant available N and P versus increased stomatal conductance (*g*_s_) on photosynthesis following fire, it remains an open question whether and to what extent these two physiological mechanisms are relevant in recovery after frequent burning.

We contrasted these physiological mechanisms leading to accelerated post-burn regrowth: (i) the root:leaf area imbalance-induced boost in water availability that top-killed plants receive, leading to reduced stomatal limitations, and (ii) the release of photosynthesis from nutrient limitation as a result of increased N and P availability with ash added to the soil surface by fire. Our main hypothesis was that top-killed plants after high-severity burn (HB) have enhanced photosynthetic rates (higher light-saturated net photosynthesis, *A*_net_) both as a result of (i) increased nutrients, especially P availability, which reduces nutrient limitation of photosynthesis and (ii) increased root:leaf area imbalance, which enhances water availability and reduces the stomatal limitations to photosynthesis. Understanding which mechanisms enable plant recovery post-fire is important for understanding species compositional changes due to fire, and for informing long-term management strategies related to prescribed burning interval and frequency.

## Materials and methods

### Sites and species

We investigated the importance of these two hypothesized post-fire photosynthetic enhancement mechanisms for plant species in eastern suburbs *Banksia* scrub (ESBS) at North Head in Manly (Sydney) NSW, Australia (33° 49' S, 151° 18' E) following a recent high-severity wildfire, which started after a prescribed burn broke through containment lines. North Head is one of a set of rocky headlands along the southeastern Australia coastline that is surrounded by sea cliffs and topped by highly leached aeolian sands (Benson and Howell 1994). The headland receives an average annual precipitation of ~1180 mm year^−1^ based on the closest and most similar weather station data from the Bureau of Meteorology (BOM station ID# 66126, data averaged across the last 25 years). North Head covers an area of 385 ha (Lambert and Lambert 2015) and contains vegetation that conforms to the critically endangered ecological community (ESBS). It is also a fire-dependent ecosystem with an 8 to 15 year fire-return time (Lambert and Lambert 2015) so it is a suitable system to investigate the ecophysiology of fire recovery by resprouting. As a result of being a prominent natural reserve and national park area in greater Sydney, Australia, there are good records of fire events dating back more than 90 years (NSW Department of the Environment 2010).

**Figure 1:**
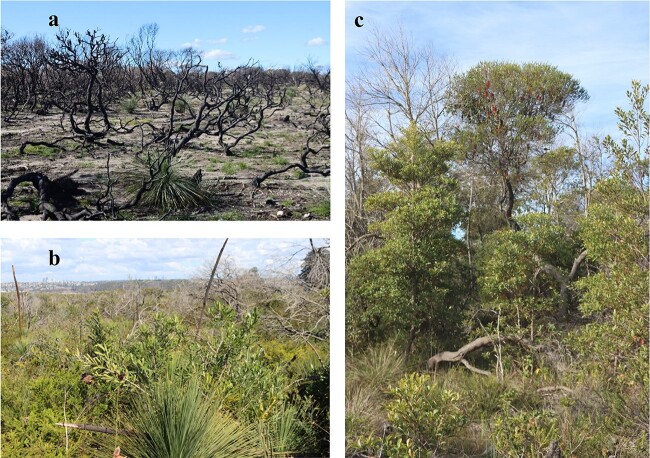
Examples of the burn type used in this study at north head, Sydney, NSW. (a) Nearly complete loss of above ground vegetation at 14 months after burning, with only *Xanthorrea* sp. seen with re-growth in an area that experienced a high-severity fire. We classed this as a HB. (b) Presence of live above-ground biomass following low-severity prescribed burns in 2012 to 2013 documented by Lambert and Lambert (2015) was considered a low-severity burn (LB). (c) Areas with no burn recorded in the last century in the DPIE fire history dataset (NSW Department of the Environment 2010) were classed as no burn (NB). These NB areas had considerable litter and organic-matter build-up over the mineral soil with shrub species and low-stature tree species dominating the canopy.

Approximately 87 ha of the headland burned by an intense wildfire on 17 to 18 October 2020. Most of the area burned with high-severity fire and combusted all live standing vegetation and ground litter in the burned zone (Fig. 1). Pockets of moderate severity burn, documented in Lambert and Lambert (2015), as well as unburned areas for the past 100 years, remained on the headland on the same soil and provided a unique ‘found’ experiment for contrasting areas of different burn severities. However, we also recognize the limitation inherent to burns that originate from different ignition sources, different severities and spatial configurations, and that occurred in different years. Three ‘burn types’ were identified across the headland, which we assigned to HB, lightly burned (LB) and no-burn (NB) classes. Similar to recent work by Tepley et al. (2018), we identified two areas as HB when a near-complete loss of aboveground vegetative structure was observed following fire (Fig. 1a), here specifically during the 2020 burn. An area classed as LB was defined by the presence of live above-ground biomass following fire (Fig. 1b) (West et al. 2016). This area had recently experienced a low-severity controlled burn from 2012 to 2013 (7 years prior to sampling) documented by Lambert and Lambert (2015). By virtue of being controlled, the areas did not experience severe burning that fully top-killed plants in this location. The low-severity burned area had extensive regrowth vegetation with low stature (<5 m tall), a thin surface layer of organic matter (<0.5 cm), and inconsistent litter coverage with exposed soil more common than coverage by organic matter. We identified two areas as unburned in recent decades (‘NB’) based on the NSW Department of Planning, Industry, and Environment’s (DPIE) fire history map dataset (NSW Department of the Environment 2010), which had no burn recorded for these areas dating back to 1920 (Fig. 1c). These areas had considerable organic-matter build-up over the mineral soil with a canopy layer dominated by shrub species and some tree species (up to roughly 7 m tall).

To test for effects of burn severity on stomatal and non-stomatal factors on the photosynthesis of species post-fire, three lignotuberous plant species common to all burn types were selected for photosynthetic measurements and leaf chemistry. These were *Banksia aemula* R. Br., *Elaeocarpus reticulatus* Sm. and *Glochidion ferdinandi* (Müll. Arg.) F.M. Bailey. Additionally, *Lambertia formosa* Sm., was sampled for leaf chemistry only as logistic constraints limited replication of gas exchange measurements in areas of high burn severity for analysis. The two Proteaceae species (*Banksia* and *Lambertia*) represent a major Australian plant family, which generally occurs amongst fire-prone and low P ecosystems that traverse the east coast of Australia (Beadle 1966). The presence of cluster roots in most Proteaceae species has a key functional role in P uptake, which might be stimulated after burning (Lambers et al. 2015; Lambers et al. 2022). The other two species present and studied, *Glochidion* and *Elaeocarpus*, are considered to be rainforest species and occur in gullies and wet heathlands near the coast, as observed on North Head. Species selection within LB and NB areas was determined by the species that were present and regrowing in HB areas at the time of sampling.

To quantify the release of leaves from P limitation due to ash-induced P enrichment following fire, total leaf P concentration was measured on all species. To identify the effect of any increase in leaf P concentration on photosynthesis (*A*_net_), controlled photosynthetic–CO_2_ response curves (*A*_net_–*C*_i_ curves) were performed on all species (see below for more details). By measuring *A*_net_–*C*_i_ curves stomatal, and non-stomatal influences on photosynthesis can be determined (Farquhar and Sharkey 1982). Leaves were also analysed for total N and C concentrations as these elements are sensitive to fire frequency and intensity (Li et al. 2021).

### Photosynthetic measurements

Leaf net photosynthesis (*A*_net_) was measured on each species across all sites in the field using Li-Cor 6400XT portable gas exchange systems (Li-Cor Inc., Lincoln, NE, USA). Photosynthetic measurements were made over 4 weeks in late spring of 2021 at ~1 year and 7 years post-fire for HB and LB, respectively, between 9 AM and noon to avoid midday depression of photosynthesis. For all plants, one sunlit and fully expanded leaf was measured *in situ*. Leaves were measured for net photosynthesis using a stepwise set of CO_2_ concentrations and under a constant, saturating photon flux density (1800 μmol photons m^−2^ s^−1^) and constant temperature (target of 25 °C) as outlined in Ellsworth et al. (2004). Relative humidity was enhanced in the leaf chamber to a target of close to 70% before and during the *A*_net_–*C*_i_ curves using hydrated soda lime. The humidities achieved averaged 72%, 69% and 69% for *B. aemula*, *E. reticulatus* and *G. ferdinandi*, respectively, and corresponded to a mean leaf-air vapour pressure difference (D_air_) of 1.29 kPa with D_air_ never exceeding 1.9 kPa. The curve-fitting of data from these photosynthetic CO_2_ response curves used the least-squares minimization approach employed in the *plantecophys* package (v.1.4–6; Duursma 2015) using R 4.1.2 (R Core Team 2022). We extracted the net photosynthetic rate at ambient conditions and current CO_2_ levels (400 μmol mol^−1^) with controlled light and temperature (here termed *A*_net_), as well as the maximum rate of carboxylation (*V*_cmax_) and the maximum electron transport supporting ribulose-diphosphate regeneration (*J*_max_) in photosynthesis (see Farquhar et al. 1980). These two variables (*V*_cmax_ and *J*_max_) represent photosynthetic capacity and are key for understanding how plant gas exchange is regulated by nutrients and water in post-fire recovery (Cernusak et al. 2006; Sharkey et al. 2007; Renninger et al. 2013). For all species except *L. formosa*, between three and five *A*_net_–*C*_i_ curves were measured on different individuals at each burn type. The measured leaf was collected with adjacent leaves of the same age class from the same or closest branch or branchlet to ensure enough tissue for chemical analyses. Leaves were placed in zip lock polyethylene bags, labelled and stored over ice prior to further processing and chemical analysis in the laboratory. Only leaf chemistry and morphology were recorded for *L. formosa*.

### Analyses of leaf structure, chemistry and soil phosphorus concentrations

Fresh leaf samples were scanned for surface area, thickness was measured using digital callipers (Mitutoyo 150 mm Digital Caliper, Mitutoyo Asia Pacific, Singapore), and then freeze-dried (> 48 h) and weighed for leaf mass per area ratio (LMA; Figure S1 available as Supplementary data at *Tree Physiology* Online). For *L. formosa*, site-average LMA values were used when calculating area-based nutrient concentrations for individuals from HB and LB areas due to lack of data. The sample LMA was calculated as the ratio of leaf dry mass (g) per leaf area (m^2^) and this was then used to convert mass-based N and P concentration to area-based units. The total N and C concentration of leaves was measured using a LECO C-N analyser (LECO model CHN828, MI, USA). Total P concentrations were determined using a Kjeldahl acid digest with concentrated sulphuric acid (99.99%) followed by digestion of leaves in a microwave digester (Speedwave, Berghoff Instruments, Eningen, Germany) with a small amount of hydrogen peroxide, the same method described in detail in Crous et al. (2015). Following digestion, samples were diluted with deionized water and concentrations were determined using the SEAL AQ2 discrete flow autoanalyser (SEAL model AQ270, Milwaukee, WI, USA). Both analyses were performed on three to five independent samples for each species at all burn types.

To test for an increased availability of P from the recent fire at North Head, we collected soil samples from the HB and NB sites and analysed these for extractable P concentrations using the Bray-I procedure (Bray and Kurtz 1945). We did not collect samples from the LB site because it was expected that most P liberated by fire would quickly become bound in the vegetation (Butler et al. 2018). Samples were collected from five subplots at both HB and NB sites, kept on ice for transportation and frozen until air dried. The extractant in this method was a dilute hydrochloric acid and ammonium fluoride solution recommended for neutral and acid soils. The method quantifies soluble P, which is regarded as the readily available form taken up by plants (Certini 2005).

### Data analyses and modelling

All analyses were performed in R 4.1.2 (R Core Team 2022). Differences in photosynthetic capacity (*A*_net_, *V*_cmax_ and *J*_max_), leaf chemistry (total area-based leaf N and P; N_area_ and P_area_), and indicators of plant water status (*C*_i_/*C*_a_, intrinsic water-use efficiency (iWUE), and *g*_s_) as a function of burn severity, species, and their interaction were tested using 2-way ANOVAs with ‘burn severity’ and ‘species’ as fixed effects in the model. We considered individuals within burn severity as replicates in our study and used a conservative *P*-value cut-off of *P*<0.01 in statistical tests of main effects. Sample sizes were *n* = 4 in all cases except unburned *Banksia* (*n* = 5) and *Elaeocarpus* (*n* = 3). Based on the nature of the 2020 burning, the individuals we measured were scattered but in closer proximity to one another within burned areas (<50 m) than were the different burn types (560 to 630 m from one another). The *C*_i_, *C*_a_, and *g*_s_ were calculated from the *A*/*C*_i_ curve at ~ 400 ppm CO_2_. Intrinsic water-use efficiency (iWUE) was calculated by dividing *A*_net_ by *g*_s_ at ~ 400 p.p.m. CO_2_. All variables were normally distributed except *g*_s_, which was log_10_ transformed prior to analysis to attain normality. Post hoc pair-wise comparisons of group means were computed using *emmeans* (v1.7.2) for one tail of the distribution, using the Tukey honestly significant difference (HSD) method of *P*-value adjustment. All regressions were fit using the least-squares approach. Two-tailed Student’s *t*-tests were performed to compare the organic matter content, pH, total N (%), total P (mg kg^−1^) and extractable P (mg kg^−1^) of the top 10 cm of soil across HB and NB sites. Organic matter, total P and extractable P were log_10_ transformed prior to analysis to attain normality.

Stomatal limitations for the species and burn treatments were assessed using the approach described by Grassi and Magnani (2005), which analysed limitations in the supply of CO_2_ to *A*_net_ by diffusion through the stomata to the intercellular spaces but assumed infinite mesophyll conductance. The approach for stomatal limitation computations is summarized in Supplementary data available at *Tree Physiology* Online (Figure S3). Photosynthesis was modelled using a simple coupled stomatal-leaf gas exchange model (Farquhar et al. 1980; Farquhar and Sharkey 1982; Duursma 2015), which used the measured *g*_s_ amongst the species and treatments and computed *A*_net_ on the basis of the intersection between the supply of CO_2_ by diffusion through the stomata, and the biochemical demand for CO_2_ by Rubisco enzyme and ribulose 1,5 bisphosphate (RuBP) regeneration (Farquhar et al. 1980). Simulations of *A*_net_ using the *plantecophys* package reproduced the measurements in linear regression with a significant slope of 1.03 ± 0.07 (*r*^2^ = 0.917). Using this modelling approach, we could determine the apparent biochemical response of post-burn by replicating the *V*_cmax_ and *J*_max_ of the post-burn measurements, but the stomatal conductance of unburned plants and vice-versa was used to evaluate the stomatal response of burning. The measured burning responses and the modelled components of the overall responses (viz. biochemical or stomatal) were evaluated by species relative to the unburned measured *A*_net_ in the unburned condition. All figures were produced using *ggplot2* (v3.3.5; Wickham 2016).

## Results

### Gas exchange of regrowth and unburned plants

**Figure 2:**
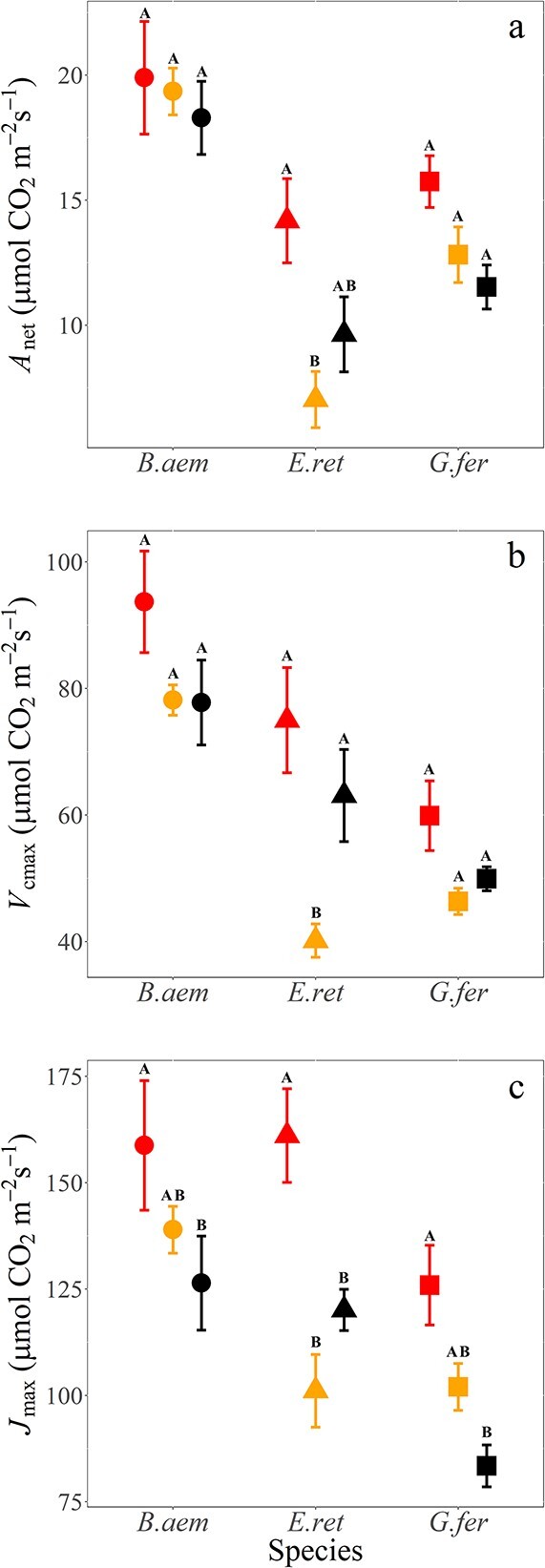
Measures of leaf photosynthetic capacity of study species across three burn types. (a) Area-based light-saturated photosynthesis (*A*_net_) (μmol CO_2_ m^−2^ s^−1^), (b) maximum rate of area-based carboxylation (*V*_cmax_) (μmol CO_2_ m^−2^ s^−1^) and (c) maximum rate of area-based RuBP regeneration (*J*_max_) (μmol CO_2_ m^−2^ s^−1^). Study species are *B. aemula*, *E. reticulatus* and *G. ferdinandi*. Within species, burn types are indicated in order of high-severity burn (HB; left-most point), lightly burned (LB) and long-unburned (NB; black points to the right). Data represent mean and ± standard error (SE). Letters denote statistically significant differences within species across treatments.

We first investigated the general patterns of plant gas exchange in post-fire recovery and unburned vegetation. We found a significant main effect of burn severity on the *A*_net_ of regrowing vegetation (*F*_2,4_ = 6.55, *P* = 0.005, Fig. 2a, Table 1), as well as for the biochemical underpinnings of photosynthesis *V*_cmax_ (*F*_2,4_ = 14.05, *P* < 0.001, Fig. 2b, Table 1) and *J*_max_ (*F*_2,4_ = 15.55, *P *< 0.001, Fig. 2c, Table 1). Across species, average rates of *A*_net_, *V*_cmax_ and *J*_max_ increased by 26.3%, 19.8% and 35.1%, respectively, in areas of high burn (HB) compared with unburned vegetation (NB) (Fig. 2, Table S1 available as Supplementary data at *Tree Physiology* Online). Within species, both *B. aemula* and *G. ferdinandi* showed increasing *A*_net_ and *J*_max_ with shorter time since burning, consistent with the overall trend from unburned to LB to HB. Contrary to this trend and apart from the other species, *E. reticulatus* consistently showed the lowest rates for all three parameters (*A*_net_, *V*_cmax_ and *J*_max_) in LB areas (Fig. 2, Table S1 available as Supplementary data at *Tree Physiology* Online). However, for *E. reticulatus*, HB still stimulated increases of 47.1%, 18.9% and 34.1% on average for *A*_net,_*V*_cmax_ and *J*_max_, respectively, in comparison with unburned vegetation. The species *A*_net_ responses to HB were + 9% for *B. aemula*, + 47% for *Elaeaocarpus* and + 36.5% for *G. ferdinandi*, respectively.

**Table 1:** Summary of two-way ANOVA for photosynthetic capacity (*A*_net_) and its biochemical determinants (*V*_cmax_ and *J*_max_) across the three species, i.e. *B. aemula*, *E. reticulatus* and *G. ferdinandi* (‘species’ in ANOVA), at North Head across three different burn regimes in the study (‘burn severity’).

**Variable**			***A***_**net**_			***V***_**cmax**_			***J***_**max**_	
	***df***		***F***	***P***		***F***	***P***		***F***	***P***
Burn severity	2		6.49	0.0047		12.55	0.00012		15.62	<0.0001
Species	2		32.67	<0.0001		27.69	<0.0001		12.15	0.00015
Burn severity × Species	4		1.65	0.189		1.81	0.153		1.85	0.146
Residual mean square error	29		8.062			121.4			365.0	

The ANOVA results shown (*F*-statistics and *P*-values) are from type II sums of squares.

### Leaf chemistry

**Figure 3:**
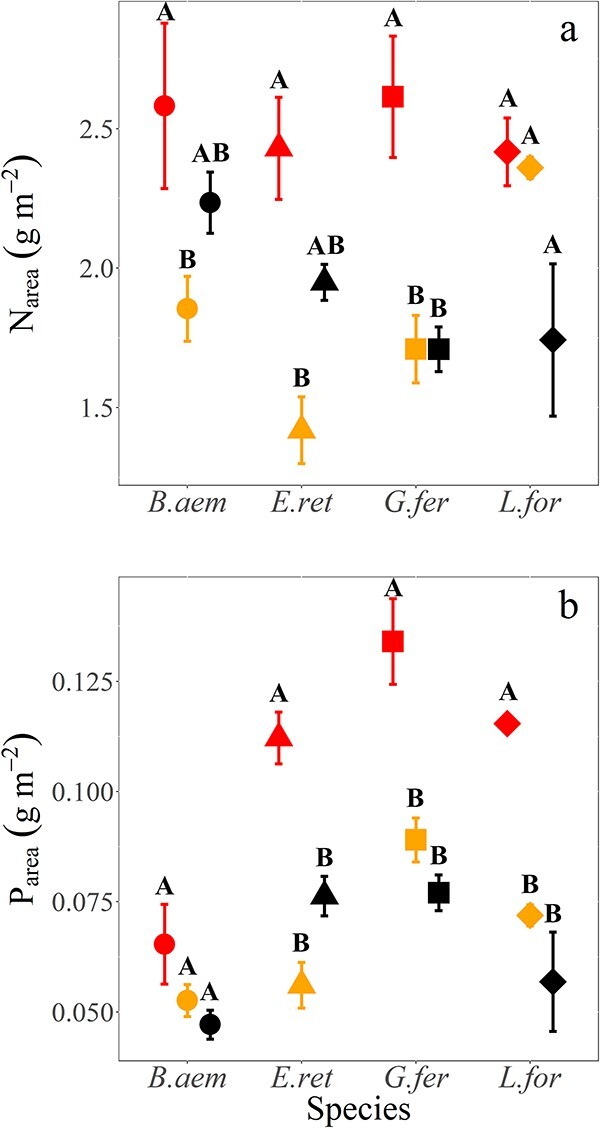
(a) Total leaf N (N_area_) (g m^−2^) and (b) total leaf P (P_area_) (g m^−2^) of study species across three burn types. Study species are *B. aemula*, *E. reticulatus*, *G. ferdinandi* and *L. formosa* and burn types are indicated as in Figure 2. Data represent mean and ± SE. Letters denote statistically significant differences within species across treatments.

Between the HB and NB locations, there were differences in soil chemistry (Table 2) with higher soil organic matter in NB and higher total N and P concentrations but lower extractable P concentrations (Table 2). The trends in leaf chemistry were expected to follow those observed for leaf gas exchange if there was strong control of nutrients over photosynthesis in this nutrient-poor ecosystem. Across species, area-based leaf N (N_area_) and area-based leaf P (P_area_) were significantly enhanced by burn treatment (*F*_2,6_ = 22.91, *P*<0.001, Fig. 3a, Table 3 and *F*_2,6_ = 49.16, *P*<0.001, Fig. 3b, Table 3 for N_area_ and P_area_, respectively), as was observed for photosynthetic parameters (Tables 1 and 3). On average and across the three species we measured for both gas exchange and leaf chemistry, burning in HB areas resulted in increases of 31% and 53% for N_area_ and P_area_, respectively, in comparison with the corresponding values for unburned vegetation. The corresponding values across the four species in Fig. 3 were slightly larger increases of 31.5% and 66% for N_area_ and P_area_, respectively. There was a significant interaction between burn severity and species for P_area_ (*F* = 2.20, *P*=0.005, Table 3), but not for N_area_ (*P*>0.05, Table 3).

**Table 2:** Physical and chemical description of the top 10 cm of mineral soil from high severity burn areas (HB) compared with areas with no previous burn in the past few decades (NB).

**Characteristic**	**HB**	**NB**
Organic matter (OM) %^**^	3.13 (0.1)	6.35 (0.4)
pH	4.83 (0.02)	4.8 (0.05)
Total N (%)^**^	0.06 (0.02)	0.12 (0.01)
Total P (mg kg^−1^)	19.48 (1.09)	58.18 (19.03)
Extractable P (mg kg^−1^)^**^	3.50 (0.74)	0.95 (0.12)

Bray-P refers to the extractable P concentration following Bray and Kurtz (1945). Data represent averages (*n* = 5 for each of the plot types) with ± standard errors (SE) shown in parentheses. If indicated, the asterisks by each soil characteristic denotes statistically significant difference between HB and NB areas where ** indicates *P*<0.01.

**Table 3:** Summary of two-way ANOVA for total leaf N (N_area_) and total leaf P (P_area_) across four study species, *B. aemula*, *E. reticulatus*, *G. ferdinandi* and *L. formosa* (‘species’ in ANOVA) at North Head across three different burn regimes in the study (‘burn severity’).

**Response**			**N**_**area**_			**P**_**area**_	
	***df***		***F***	***P***		***F***	***P***
Burn severity	2		22.77	<0.001		49.16	<0.001
Species	3		2.08	0.121		27.06	<0.001
Burn severity × Species	6		2.20	0.066		3.84	0.005
Residual mean square error	36		0.114			0.0002	

*F*-test results use type II sums of squares.

Across species, a broad pattern of decline in leaf nutrient contents (N_area_ and P_area_) from HB to other burn severities was observed (Fig. 3 and Burn severity main effect in Table 3). Leaf nitrogen (N_area_) was elevated in HB compared with unburned areas (NB) only for *G. ferdinandi* (*P*<0.05, Fig. 3a, Table S2 available as Supplementary data at *Tree Physiology* Online), and N_area_ decreased significantly (*P*<0.05) from HB to LB for three of the four species. There were stronger trends with regard to leaf P content, with *E. reticulatus*, *G. ferdinandi* and *L. formosa* showing significantly higher P_area_ across HB to NB areas (*P*<0.005, Fig. 3b, Table S2 available as Supplementary data at *Tree Physiology* Online for all three species). *Banksia aemula* was the only species that did not show a significant difference in P_area_ as a function of burn severity (*P*>0.05), so as a result there were highly significant Burn severity × Species interactions (*P*=0.005) for P_area_ (Table 3). The evidence supported larger responses of P_area_ to burn severity than N_area_ (Fig. 3, Table 3, and Table S2 available as Supplementary data at *Tree Physiology* Online).

**Figure 4:**
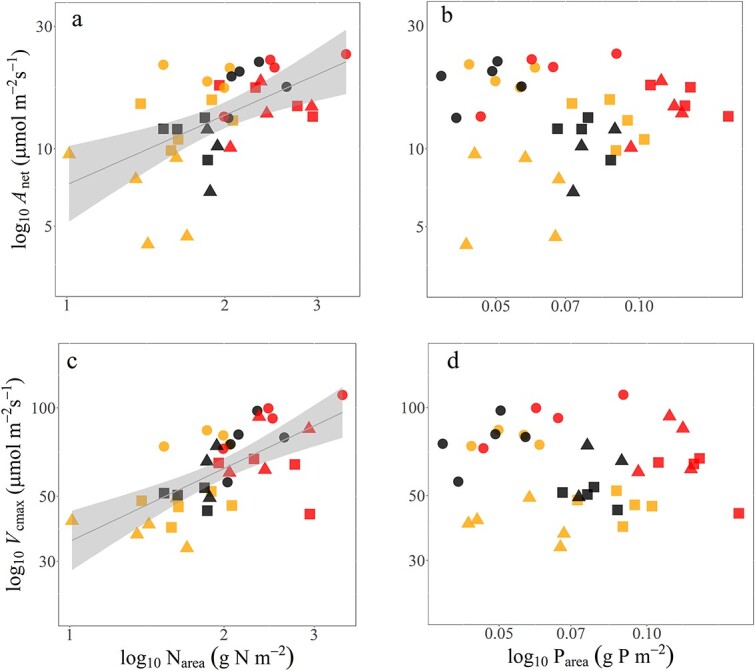
Relationship of log–log light-saturated photosynthesis (*A*_net_) with (a) total leaf N per unit area (N_area_) and (b) total leaf P per unit area (P_area_). Relationship of the maximum rate of carboxylation (*V*_cmax_) with (c) N_area_ and (d) P_area_. Symbols represent the study species where *B. aemula*, *E. reticulatus* and *G. ferdinandi* are shown as a circle, triangle and square, respectively. Burn severity is indicated as in Figure 2. The relationships in panels a and c were highly significant (*P*<0.0001). The *r*^2^ value for *A*_net_ with N_area_ was 0.29, with an *r*^2^ value for *V*_cmax_ with N_area_ of 0.42. Relationships with *J*_max_ and N_area_ or P_area_ were similar to those for *V*_cmax_ and those variables (data not shown).

We sought to establish if there was a link between the burn-induced changes in gas exchange parameters in Fig. 2 with leaf nutrients. Indeed, *A*_net_ was significantly correlated with leaf N (*P*=0.00044, *r*^2^ = 0.29) across species and burn severities (Fig. 4a). Examining V_cmax_ in this regard as a strong biochemical determinant of net photosynthesis at current CO_2_ levels, there was an even stronger relationship (Fig. 4c, *P*< 0.00001, *r*^2^ = 0.42). In contrast, neither *A*_net_ nor V_cmax_ was significantly related to leaf P content (Fig. 4b and d, *P*=0.71 and 0.52 for *A*_net_ and *V*_cmax_, respectively).

### Leaf conductance

**Figure 5:**
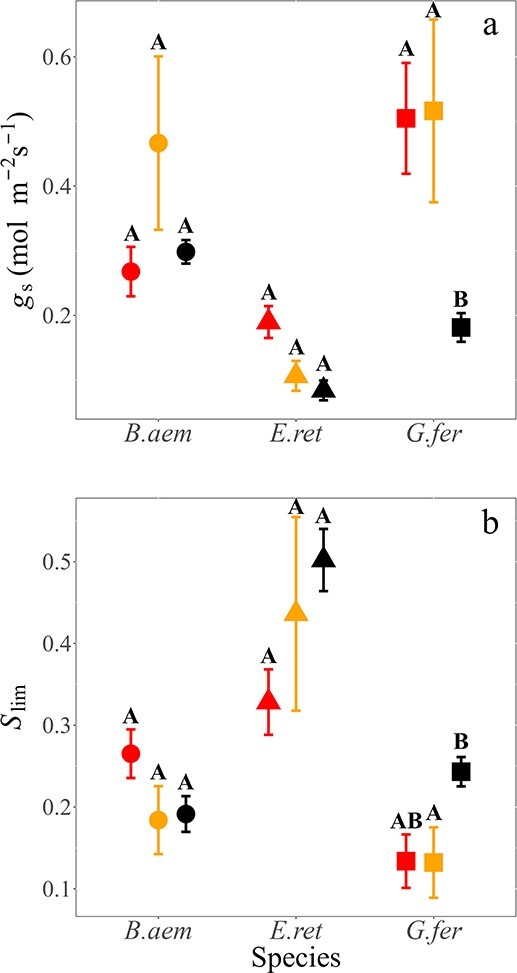
(a) Stomatal conductance (*g*_s_) of study species across three burn types and (b) stomatal limitations to photosynthesis (*S*_lim_) (unitless) across burn types. Study species are *B. aemula*, *E. reticulatus* and *G. ferdinandi*, with symbols as indicated in Figure 2. Data represent means ± SE. Letters denote statistically significant differences within species across treatments.

We examined three key gas exchange indicators related to the conductance to water vapour for recent burn resprouts in HB areas compared with LB and unburned areas: *g*_s_, *C*_i_/*C*_a_ ratio and stomatal limitations to *A*_net_ (*S*_lim_). Across species, burn severity did not significantly affect *g*_s_ (*P*=0.060, Fig. 5a) or *S*_lim_ (*P*=0.11, Fig. 5b), but there were significant Burn severity × Species effects (Table 4). Specifically contrasting HB and unburned areas, *G. ferdinandi* showed significantly higher *g*_s_ values in HB (*P*=0.01), while *g*_s_ for *E. reticulatus* was not significantly higher (*P*=0.06). There were significant species differences in both *S*_lim_ and *C*_i_/*C*_a_ ratio (*P*<0.001, Fig. 5, Table 4, and Fig. S2 and Table S3 available as Supplementary data at *Tree Physiology* Online) relating to differences in stomatal regulation and the setpoint to gas exchange.

**Table 4:** Summary of two-way ANOVA for the *g*_s_ and stomatal limitations to *A*_net_ (*S*_lim_) across the three main study species and three different burn regimes as in Table 2.

**Variable**		***g***_**s**_		***S***_**lim**_	
	***df***	***F***	***P***	***F***	***P***
Burn severity	2	4.66	0.0175	2.39	0.1093
Species	3	23.23	<0.0001	7.63	0.00011
Burn severity × Species	6	2.84	0.0423	1.69	0.169
Residual mean square error	36	0.210	0.170		

*F*-statistics are computed using type II estimates.

### Control of photosynthesis

**Figure 6:**
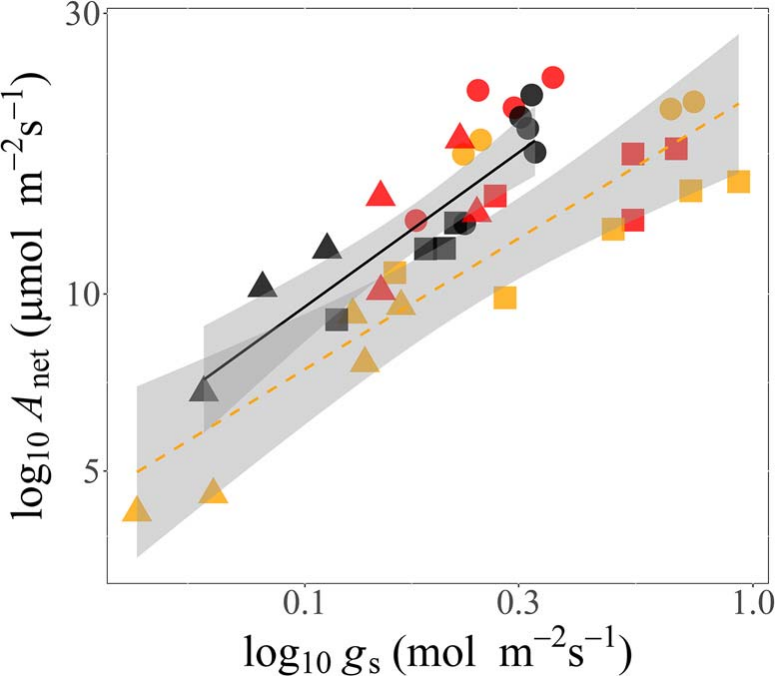
Relationship of log–log area-based light-saturated photosynthesis (*A*_net_area_) (μmol CO_2_ m^−2^ s^−1^) with stomatal conductance (*g*_s_) (mol m^−2^ s^−1^). Symbols represent study species where *B. aemula*, *E. reticulatus* and *G. ferdinandi* are shown as a circle, triangle, and square, respectively. Burn severity as high burn severity (HB), light burn severity (LB) and an area with no recent burn (NB), respectively are indicated as in Figure 2. Regression lines were fit to statistically significant relationships according to burn type, with the solid line indicating NB, the dashed line indicating LB, and HB was not significant. Adjusted *r*^2^ values for *A*_net_area_–*g*_s_ relationships were 0.82, 0.73 and 0.05 for NB, LB and HB, respectively.

**Figure 7:**
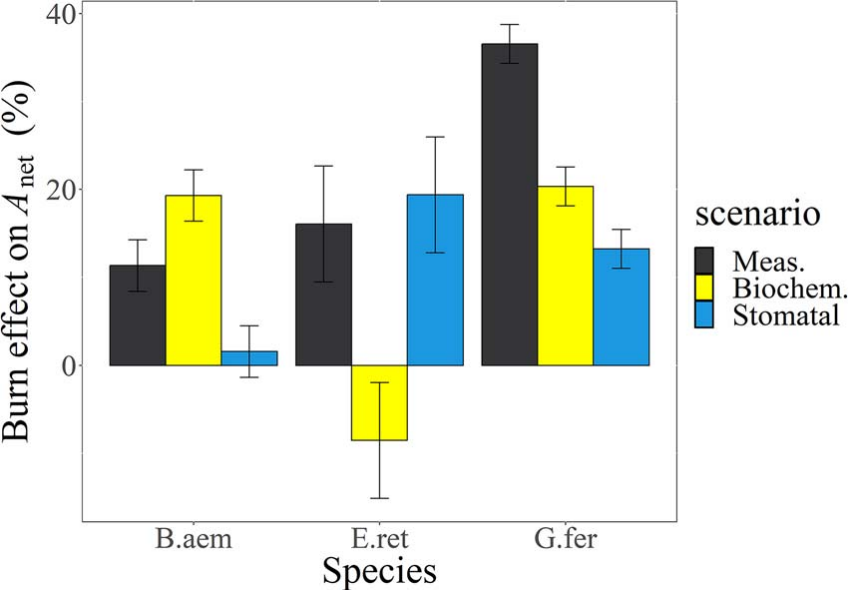
Modelling analysis of key mechanisms responsible for the post-fire increase in photosynthesis, generated using a coupled stomatal-leaf gas exchange model. ‘Meas.’ indicates the measured burn effect on *A*_net_, ‘Biochem.’ refers to the stimulation of leaf nutrients leading to adjustment in the rates associated with the biochemistry of photosynthesis and ‘Stomatal’ refers to the increase in stomatal conductance and *C*_i_/*C*_a_ as the set-point for leaf *A*_net_. Error bars are the standard error of the mean among individuals.

There was a strong relationship between stomatal conductance and photosynthesis across species and burn treatments, particularly in unburned areas (Fig. 6). However, both rainforest species *G. ferdinandi* and *E. reticulatus* showed low *A*_net_ per unit *g*_s_ with burning as evidenced by data falling outside the 95% confidence interval (CI) for the relationship across species in non-burned areas (coloured triangles and squares in Fig. 6). This analysis suggested reductions in iWUE with burning for these species, which led us to examine the controls over *A*_net_ elicited by both biochemical and stomatal regulation after burning using a modelling approach (Fig. 7).

Modelled *A*_net_ closely matched the measured *A*_net_ across species and burn treatments (slope = 1.03, *df* = 21, *r*^2^ = 0.92, *P*<0.00001; data not shown), so we had sufficient confidence to undertake a modelling analysis of controls over *A*_net_. For the modelled burn effect on *A*_net_, the burn effect size we observed for *B. aemula* was similar to the modelled biochemical effect (11% versus 19%; Fig. 7). There was almost no difference in *g*_s_ between burned and unburned plants of *B. aemula* (Fig. 5a), hence no stomatal effect on *A*_net_ was observed. For *E. reticulatus*, the burn effect on *A*_net_ that we observed (16%) was the opposite: the stomatal effect computed at 19%, which closely replicated the observations (Fig. 5a). Finally, for *G. ferdinandi*, the burn effect size of 37% was approximately the sum of the modelled biochemical effect (20%) and a 13% stomatal effect on *A*_net_.

## Discussion

Post-fire increases in leaf net photosynthesis like those we observed (Fig. 2) serve to re-establish whole-tree biomass following resprouting on sites where severe-intensity burns have removed most aboveground biomass (Clarke et al. 2013). The mode of plant response to this burning has important implications for management and fire return frequencies. Whether increases in foliar nutrients or leaf-level transpiration are the principal physiological mechanism to support increased growth has long been debated (Castell et al. 1994; Nolan et al. 2021). Here we found strong increases in leaf P_area_ with increasing burn severity (Fig. 3b) and statistically weaker but still significant increases in leaf N_area_ as well (Table 3, Fig. 3a). Several syntheses have also found that soil N concentrations can be enriched for one to several years after a fire (Johnson and Curtis 2001; Certini 2005; Pellegrini and Jackson 2020). There are also examples of soil P enhancement in post-fire landscapes (see Butler et al. 2018). Soils across a burned landscape can be varied in their N and P availability (Chambers and Attiwill 1994). However, soils under burned vegetation typically show increased concentrations of inorganic P (P_i_) (Adams et al. 1994; Chambers and Attiwill 1994; Escudey et al. 2010), which has been shown to steadily increase with increasing fire temperature (Chambers and Attiwill 1994) and hence fire severity. As P_i_ is more readily available for uptake by plants than organic P (Certini 2005; Dijkstra and Adams 2015; Schaller et al. 2015), the increase in P_i_ would drive increases in P_area_ after burning. Together with increases in soil N and other macronutrient elements (Butler et al. 2019), the increases in P lead to a situation where plants should experience enhanced nutrient availability due to post-fire impacts on soil chemistry, at least in the aftermath of severe burning (see Fig. 3). The results from the light-burn intensity have implications for the intensity of controlled burns in high-intensity-adapted vegetation like this coastal shrubland.

The increases in leaf N_area_ with burning in Fig. 3 were significantly correlated with *A*_net_ and *V*_cmax_ (Fig. 4), and *J*_max_ as well (data not shown). In support of the significant increases in *A*_net_ and photosynthetic capacity (*V*_cmax_ and *J*_max_) we observed across species as a result of burning (*P*<0.001; Fig. 2, Table 1), other studies have also observed similar post-burn increases in photosynthesis (Reich et al. 1990; Clemente et al. 2005; Utsumi et al. 2010; Goorman et al. 2011; Turnbull et al. 2014). There are far fewer results involving the biochemical parameters for photosynthesis, although Renninger et al. (2013) did show a strong burn effect on *V*_cmax_. Given the ability of nutrients to strongly limit photosynthetic capacity (Reich et al. 2009; Ellsworth et al. 2022), it is unsurprising that plants readily capitalize on the transient boost in available N and P in soils, particularly in the immediate growing season post-fire. However, an enhancement in photosynthetic rates and capacity following fire is not reported across all studies, with some reporting little or no enhancement of photosynthetic rates (Cernusak et al. 2006; Renninger et al. 2013). It is important to take account of burn severity and time since burning in these analyses given the lack of consistent differences that we observed for N_area_ and P_area_ in the four species we studied across LB to NB areas (Fig. 3). A potential limitation of our study was the unplanned or ‘found’ experimental design that limits replication for both burn severity and time since burning, and the lack of before-versus-after burning data. However, planned replication of the severity of burn that was achieved over tens of hectares is highly unlikely in a peri-urban context such as in the Sydney basin, so the insights from our study provide insight into the mechanisms of plant response in this fire-prone coastal system.

Our central hypothesis was that post-fire resprouting plants would have enhanced photosynthetic rates in comparison to mature, unburned plants as a result of a transient release from both stomatal limitations and nutrient (leaf P and N) constraints on photosynthesis. This was only partially supported by our results. Although P_area_ showed strong increases related to burn severity (nearly 50% increase across the four species, Fig. 3b), increases in this nutrient were not strongly connected to our measures of photosynthesis or biochemistry (Fig. 4b and d). In contrast, the burn-related increases in N_area_ ranged from 15% to 45% across species (Fig. 3a) and were linked to increases in photosynthesis (Fig. 4a and c). Leaf N has been found to be a strong correlate of photosynthetic capacity in plants due to the predominance of ribulose-bisphosphate carboxylase/oxygenase (Rubisco; Luo et al. 2021), the primary photosynthetic enzyme to which a large proportion of leaf N is allocated, while the functional role of P in regulating photosynthesis remains less clear (Ellsworth et al. 2022). The strong correlation of the photosynthetic–nutrient relationships suggests that burn-induced increases in soil nutrients and nutrient cycling could have a more significant role in boosting vegetative regrowth than a boost in water loss in exchange for carbon gain in low-P heathlands and scrublands like those in our study (Handreck 1997). Still, the modelling analysis in Fig. 7 indicated that in the rainforest species a component (for *G. ferdinandi*) or nearly all (for *E. reticulatus*) of their photosynthetic responses could be attributed to increased stomatal conductance.

In addition to biochemical influences that increase *A*_net_ through changes in leaf chemistry and increased incorporation of N into enzymes, further *A*_net_ increases can occur as a result of enhanced *g*_s_ with an excess of water-absorbing roots after top-kill by burning. Post-fire top-kill of woody plants increases transpiration rates of resprouting leaves and facilitates their high rates of gas exchange through reduced stomatal limitations. The subsequent *A*_net_ increases can enable the relatively rapid recovery of leafy material towards pre-fire levels (Castell et al. 1994; Clemente et al. 2005; Utsumi et al. 2010; Nolan et al. 2014). In resprouting leaves, the increase in transpiration rates occurs with a *g*_s_ increase in burned plants as an increased root:shoot ratio allows a more liberal water-use strategy in their regrowth than in unburned plants (Castell et al. 1994). Goorman et al. (2011) also found a less conservative water-use strategy was adopted by plants following fire with higher leaf water content and *g*_s_, and lower water-use efficiency than unburned plants. Although there was no statistical main or interactive effect of burning with species in our study for *S*_lim_ (Table 4), our results did show burning stimulated significantly higher *g*_s_ for *E. reticulatus* and *G. ferdinandi* when comparing HB with NB areas (*P*<0.05; Fig. 5a, Table 4, and Table S2 available as Supplementary data at *Tree Physiology* Online). This corresponded to higher *C*_i_/*C*_a_ for *E. reticulatus* and *G. ferdinandi*, respectively (Fig. S2 and Table S2 available as Supplementary data at *Tree Physiology* Online), as expected. In this analysis, some caution is warranted because the stomatal conductance measurements in our study rely only on a small number of replicates.

The results suggest a less conservative water-use strategy demonstrated by both these species after fire than before burning. In support of this, the relationship between *g*_s_ and photosynthetic rate diverged by burn type (Fig. 6), towards lower *A*_net_ for a given *g*_s_ for the two rainforest species *E. reticulatus* and *G. ferdinandi*, suggesting lower iWUE for these species. These responses are not universal and may be species-specific (Renninger et al. 2013) as shown by the Burn severity × Species interaction for *g*_s_ (Table 4). Part of the reason why the stomatal release from limitations are less effective for enhancing *A*_net_ after burning compared with changes in leaf biochemical parameters is shown by the *A*_net_– *g*_s_ decoupling in high burn (HB) sites in Fig. 6.

Assessments of both stomatal limitations and photosynthetic biochemistry for post-burn responses of woody plants are rare. We sought to attribute the post-burn resprouting leaf response of *A*_net_ to leaf biochemistry or conductance via a modelling analysis (Fig. 6). This modelling to attribute the burn response to leaf nutrients or conductance showed that both phenomena were in play and the importance of each factor for the *A*_net_ response to burning was species-dependent. Since the coupled *A*_net_-*g*_s_ model relies on C_i_ as the setpoint to determine *A*_net_, and C_i_ is recursive in that it both controls *A*_net_ but is also dependent on the *A*_net_ drawdown of CO_2_ inside leaves (Farquhar and Sharkey 1982), the overall *A*_net_ responses that we modelled (Fig. 7) may not be a simple sum of the individual responses for the scenarios for biochemistry and stomatal conductance. However, the coincidence of the modelled scenarios for *A*_net_ with the measured *A*_net_ responses to burning does suggest that the attribution modelling done here describes the two overall influences for the burn responses of these species. This lends evidence to a stronger enhancement of leaf biochemistry by increased N_area_ than by increased *g*_s_. Our results provide some insight into how woody resprouting species in fire-prone, low-P heathland communities might perform photosynthetically and point to some of the determinants of this response. Understanding these mechanisms of achieving higher *A*_net_ in resprouters after fire can be relevant to how these species may perform under increasing fire frequency, the likelihood of which is increased with warmer and drier conditions (Canadell et al. 2021).

## Conclusions

Given the large areas of forest and shrubland that is burnt each year, it is important to understand the photosynthetic mechanisms of tree regrowth after fire to understand the resilience of these systems and for management to encourage or enhance recovery. We sought to evaluate the release from stomatal limitations and biochemical limitations during burn recovery after severe fire in an Australian coastal scrub ecosystem. We found substantial increases in leaf area-based N and P by 31 and 53%, respectively. We related these increases in leaf N_area_ to the increase in photosynthesis and carboxylation capacity after fire. The modelling analyses suggested that release from stomatal limitation played a lesser role in photosynthetic increases for two of the three species studied. The post-fire photosynthetic responses to burning from nutrient release accelerate woody species recovery in the fire-prone, low-P heathland community but also suggests that possible nutrient losses by increasingly frequent prescribed burning will serve as a barrier to rapid woody regrowth in coastal heathlands.

## Supplementary Material

Rogers_Ellsworth_Supplementary_Information_tpae079

## Data Availability

The datasets generated and analyses during the current study are available at Figshare (https://doi.org/10.6084/m9.figshare.26103277.v1).
