# Supplementary material for: Stimulated photosynthesis of regrowth after fire in coastal scrub vegetation: increased water or nutrient availability?
Source: Tree Physiol. 2024 Jul 3;44(8):tpae079. doi: 10.1093/treephys/tpae079 (PMC11299026; doi:10.1093/treephys/tpae079)
Supplement: Rogers_Ellsworth_Supplementary_Information_tpae079 [file rogers_ellsworth_supplementary_information_tpae079.docx]

**Supplementary Information (SI)**

| 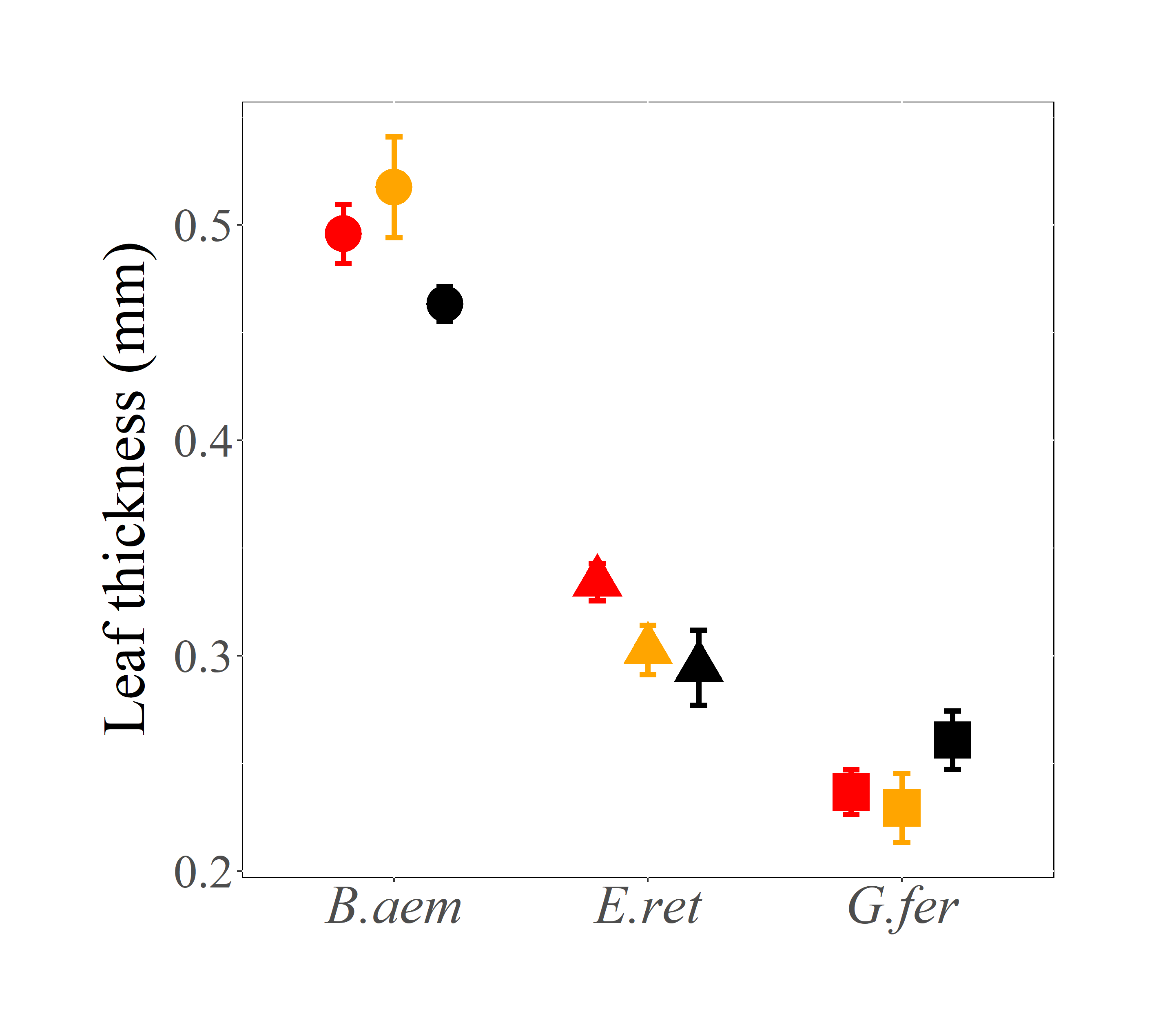  **(a)** | 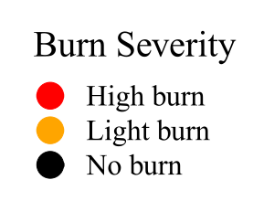 |
| --- | --- |
| 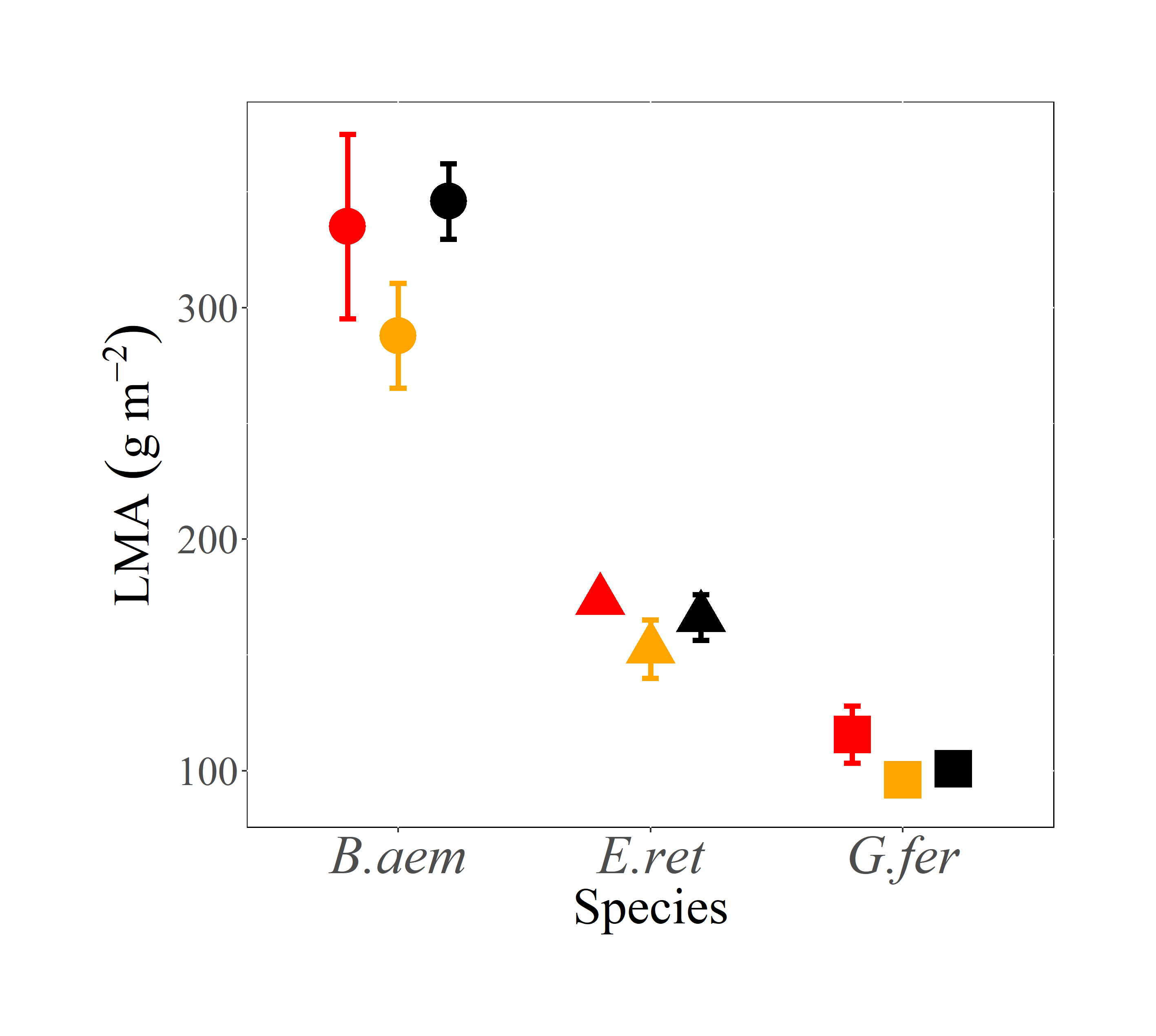  **(b)** |  |

**Figure S1 a** leaf thickness (mm), and **b** leaf mass per area (LMA) (g m^-2^) of study species across three burn types. Study species are *Banksia aemula, Elaeocarpus reticulatus,* and *Glochidion ferdinandi*. Data represent mean and ± SEM.

| **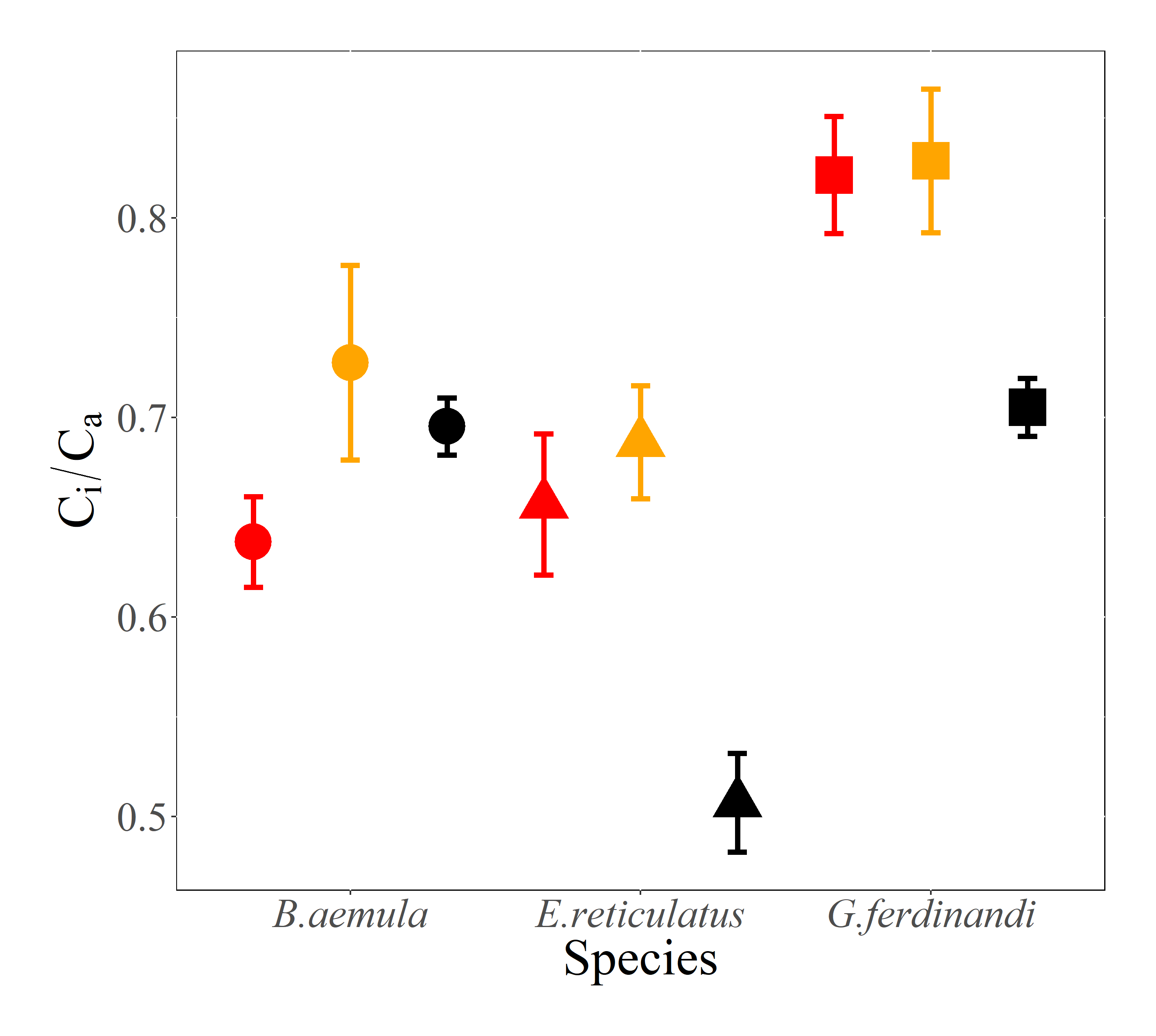** | 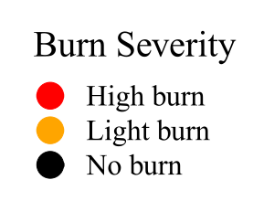 |
| --- | --- |

**Figure S2** The ratio of leaf internal to external CO_2_ concentration (*C*_i_/*C*_a_) computed from gas exchange measurements for the three study species across three burn types. Study species are *Banksia aemula, Elaeocarpus reticulatus,* and *Glochidion ferdinandi*. Data represent mean and ± SEM.

**Stomatal limitation calculations**

We used the C_i_ at ambient CO_2_ and fits to the controlled *A*_net_ – *C*_i_ curves to compute stomatal limitations to photosynthesis for each species and in their respective burn types. The approach described by Grassi and Magnani (2005) was used to compute stomatal limitations to photosynthesis. From the derivation shown in Grassi and Magnani (2005), stomatal limitations to CO_2_ exchange (*S*_lim_) can be computed by:

$$S_{lim}=\frac{\frac{g_{tot}}{g_{s\_CO2}}*\frac{{\partial A}_{net}}{\partial C_{i}}}{g_{tot}+\frac{{\partial A}_{net}}{\partial C_{i}}}$$

where:

*g*_tot_ is total conductance to CO_2_ between the outside air near the leaf surface and the internal air spaces of a leaf (e.g., 1/*g*_tot_ = 1/*g*_bl_ + 1/g_s_CO2_);

*g*_s_CO2_ is the stomatal conductance to CO_2_, computed as *g*_s_/1.6 where 1.6 accounts for differences in the diffusivities for CO_2_ and H_2_O vapour;

*A*_net_ is the light-saturated leaf net CO_2_ assimilation rate measured using steady-state gas exchange;

C_i_ is the CO_2_ mole fraction in the substomatal cavity;

and the ∂*A*_net_/∂C_i_ term is the partial derivative of *A*_net_ with respect to intercellular CO_2_ mole fraction, as illustrated in Fig. S3. As mesophyll conductance to CO_2_ was not used in our computations, the contrasting biochemical limitation to photosynthesis can be considered equivalent to: 1 - *S*_lim_.

We used the R package *plantecophys()* for the calculations by modelling *A*_net_ at different C_i_ values (Fig. S3). The modelled *A*_net_ using the measured C_i_ reproduced well the measured photosynthesis (r^2^ = 0.92 and slope = 1.03) with a slope very close to 1:1, giving confidence to the *S*_lim_ calculations.


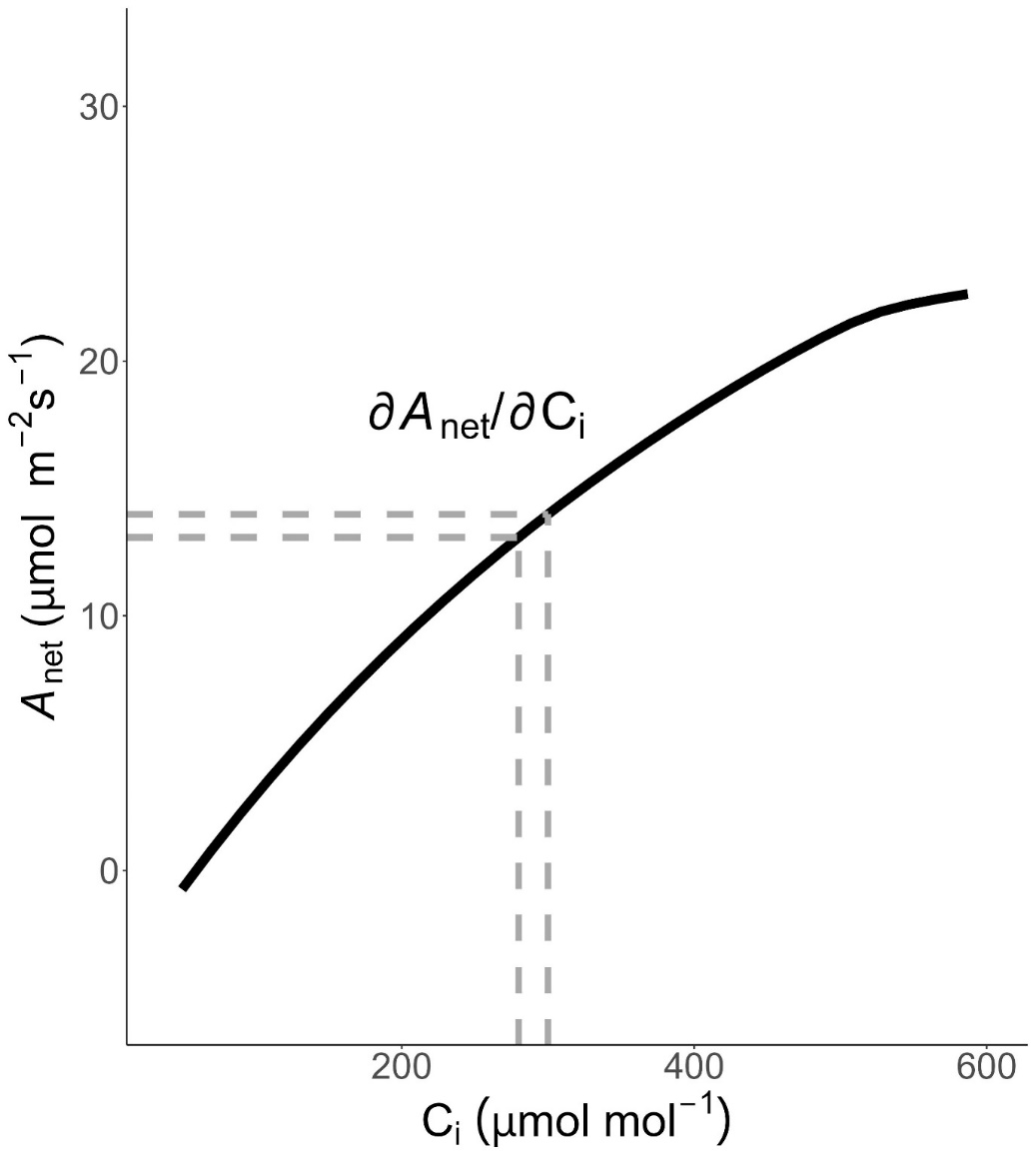


**Figure S3** Conceptual diagram for the computation of the response of *A*_net_ to C_i_ draw-down as a component of the stomatal limitation computation. The example given is for the mean *A*_net_ – *C*_i_ curve for *Glochidion ferdinandi* at the HB area. The grey lines illustrate the change in *A*_net_ for a small change in *C*_i_.

**Table S1** Outcomes from contrasts of least-squares means for photosynthetic capacity (*A*_max_, *V*_cmax_, and *J*_max_). Estimates are based on the ‘emmeans’ package, with comparisons between burn intensities: ‘HB-LB’ high versus light burn, ‘HB-NB’ high burn versus no burn, and ‘LB-NB’ light burn versus no burn.

| **Variable** |  | ***A*_max_** | | |  | ***V*_cmax_** | | |  | ***J*_max_** | | |  |
| --- | --- | --- | --- | --- | --- | --- | --- | --- | --- | --- | --- | --- | --- |
|  |  | **HB – LB** | **HB - NB** | **LB - NB** |  | **HB – LB** | **HB - NB** | **LB - NB** |  | **HB – LB** | **HB - NB** | **LB - NB** |  |
| *Banksia aemula* |  | ns | ns | ns |  | ns | ns | ns |  | ns | * | ns |  |
| *Elaeocarpus reticulatus* |  | ** | ns | ns |  | *** | ns | ns |  | *** | * | ns |  |
| *Glochidion ferdinandi* |  | ns | ns | ns |  | ns | ns | ns |  | ns | ns | * |  |

*P*-values are represented by *P < 0.05; **P < 0.01; and ***P < 0.001. When P > 0.05, n.s. denotes ‘not significant’ (P > 0.05).

**Table S2** Summary of least-squares means for leaf N and P contents (N_area_ and P_area_) computed with the ‘emmeans’ package. Comparisons between burn intensities are ‘H-N’ which contrasts high intensity versus no burn, and ‘H-L’ which contrasts high and low burn intensities.

| **Variable** | **N_area_** | | |  | **P_area_** | | |  |
| --- | --- | --- | --- | --- | --- | --- | --- | --- |
|  | **HB – LB** | **HB - NB** | **LB - NB** |  | **HB – LB** | **HB - NB** | **LB - NB** |  |
| *Banksia aemula* | * | ns | ns |  | ns | ns | ns |  |
| *Elaeocarpus reticulatus* | *** | ns | ns |  | *** | ** | ns |  |
| *Glochidion ferdinandi* | *** | *** | ns |  | *** | *** | ns |  |
| *Lambertia formosa* | ns | ns | ns |  | ** | *** | ns |  |

P-values are represented by **P < 0.01; and ***P < 0.001. When P > 0.05, n.s. denotes ‘not significant’.

**Table S3** Outcomes from contrasts of least-squares means for stomatal variables (*g*_s_, *C*_i_/*C*_a_, and *S*_lim_). Estimates are based on the ‘emmeans’ package, with comparisons between burn intensities: ‘HB-LB’ high versus light burn, ‘HB-NB’ high burn versus no burn, and ‘LB-NB’ light burn versus no burn.

| **Variable** |  | ***g*_s_** | | |  | ***C*_i_/*C*_a_** | | |  | ***S*_lim_** | | |  |
| --- | --- | --- | --- | --- | --- | --- | --- | --- | --- | --- | --- | --- | --- |
|  |  | **HB – LB** | **HB - NB** | **LB - NB** |  | **HB – LB** | **HB - NB** | **LB - NB** |  | **HB – LB** | **HB - NB** | **LB - NB** |  |
| *Banksia aemula* |  | ns | ns | ns |  | ns | ns | ns |  | ns | ns | ns |  |
| *Elaeocarpus reticulatus* |  | ns | ns | ns |  | ns | * | ** |  | ns | ns | ns |  |
| *Glochidion ferdinandi* |  | ns | ** | * |  | ns | * | * |  | ns | ns | * |  |

*P*-values are represented by *P < 0.05; **P < 0.01; and ***P < 0.001. When P > 0.05, n.s. denotes ‘not significant’.
